# Neuropsychiatric presentation of Wernicke’s Encephalopathy occurs to a pregnant Woman: A case report

**DOI:** 10.1192/j.eurpsy.2023.668

**Published:** 2023-07-19

**Authors:** S. Chebli, M. Besbes, R. Felhi, G. Hamdi, B. Paindaveine

**Affiliations:** ^1^centre hospitalier de Gonesse, Gonesse; ^2^centre hospitalier de Gonesse, Gonesse, France

## Abstract

**Introduction:**

Wernicke’s encephalopathy (WE) is a severe neuropsychiatric syndrome resulting from thiamine deficiency (vit B1) wich is often associated with chronic alcoholism. The classical presentation is characterized by ophtalmoplegia, ataxia and confusion.

Unfortunately, WE is still underdiagnosed because it may not always show up with a classical presentation in one hand, and could also be seen in other non alcoholic condition in an other hand which delay diagnosis and managment of early proper treatement

**Objectives:**

This case highlights the importance of considering atypical presentations of Wernicke’s encephalopathy, it’s medical etiologies and the importance of improving diagnosis to manage early treatement

**Methods:**

We reported a case of a pregnant women who consulted for alterated mental status , asthenia and occurs to have Wernicke’s encephalopathy due to hyperemesis gravidarum

**Results:**

Mrs X is a 35-year-old pregnant women with a past medical history of a cesarian, an hospitalisation in third month of this pregnancy for vomits, no known psychiatric illness or history of substance abuse. She was brought to the gynecology emergency department for asthenia and altered mental status. MRS X, was lethargic, had not eaten for several days, vomiting for more than a month. On the medical evaluation she appeared confused, disoriented, and unresponsive to verbal or manual redirection and prompting. She had also ataxia and gait incoordination. Laboratory testing was remarkable for lactic acidosis (blood lactate concentration lipasemia and normal electrolyte levels, cerebrospinal fluid (CSF) culture was unremarkable.

A brain MRI was done and showed FLAIR signal abnormalities around the third ventricle and periaqueductal, suggesting Gayet-Wernicke encephalopathy. Thiamine (vit B1) 500mg thrice a day was administrated for the next days in associtation with vitamin B6.

**Conclusions:**

Wernicke encephalopathy (WE) is an acute reaction to thiamine deficiency which usually presents with a classical triad. However, Clinicians tend to ignore WE in other non-alcoholic clinical settings and the diagnosis becomes even more difficult when thiamine deficiency presents with unusual neuropsychiatric signs and symptoms like catatonia. This case highlights the importance of considering atypical presentations of WE, it’s medical etiologies and the necessity of a complete medical evaluation and appropriate investigations to make prompt diagnosis and early management.

**Disclosure of Interest:**

None Declared

